# Patient-Derived Training Simulator for Image-Guided Adaptive Brachytherapy of Locally Advanced Cervical Cancers: Development and Initial Use

**DOI:** 10.3390/jcm11113103

**Published:** 2022-05-30

**Authors:** Kento Tomizawa, Takahiro Oike, Ken Ando, Daisuke Irie, Makoto Sakai, Hirofumi Shimada, Tatsuya Ohno

**Affiliations:** 1 Department of Radiation Oncology, Gunma University Graduate School of Medicine, 3-39-22 Showa-machi, Maebashi 371-8511, Gunma, Japan; m12201058@gmail.com (K.T.); ken.ando0906@gmail.com (K.A.); daisuke_i@gunma-u.ac.jp (D.I.); tohno@gunma-u.ac.jp (T.O.); 2 Gunma University Heavy Ion Medical Center, 3-39-22 Showa-machi, Maebashi 371-8511, Gunma, Japan; sakai-m@gunma-u.ac.jp (M.S.); shimada@gunma-u.ac.jp (H.S.)

**Keywords:** cervical cancer, image-guided adaptive brachytherapy, interstitial needles, training, 3D modeling, IC/IS applicator

## Abstract

Image-guided adaptive brachytherapy (IGABT) using intracavitary and interstitial (IC/IS) techniques plays a pivotal role in definitive radiotherapy for locally advanced cervical cancers. However, the training opportunities for interstitial needle application are limited, preventing this technique from becoming widespread. This study aimed to develop a training simulator for IC/IS brachytherapy. The simulator consists of a soft silicone tumor phantom and acrylic tube mimicking the vagina; it has high visibility because of translucent materials and is compatible with computed tomography (CT) and magnetic resonance imaging (MRI). A patient harboring a typical bulky and irregular-shaped cervical tumor was selected from 495 in-house IGABT-treated candidates, and a tumor phantom (68 × 49 × 45 mm) modeled on this patient was produced from three-dimensional real-scale measurements of the MRI-based high-risk clinical target volume at first brachytherapy. In trial use by two physicians with different levels of IGABT skills, a Fletcher-Suit Asian Pacific applicator, and a Venezia applicator with interstitial needles were nicely applied to the simulator, facilitating successful creation of CT-based treatment plans consistent with clinical practice. Thus, the training simulator can be useful for the training of IC/IS brachytherapy, and warrants further research employing a greater number of phantoms and practitioners to verify its educational value.

## 1. Introduction

Cervical cancer causes more than 300,000 deaths worldwide annually, and its mortality ranks fourth among all cancers [1]. Radiotherapy plays a pivotal role in the definitive treatment of locally advanced cervical cancers [2], with definitive radiotherapy approaches including external beam radiotherapy (EBRT) and brachytherapy [3]. For the latter, image-guided adaptive brachytherapy (IGABT) using computed tomography (CT) or magnetic resonance imaging (MRI) has become widespread in recent years [4,5]. The introduction of IGABT technologies into definitive radiotherapy practice has dramatically improved the oncologic outcomes of cervical cancers [6,7].

Despite the technological advancements, IGABT using a tandem and ovoid applicator alone results in suboptimal dose coverage for bulky or irregular-shaped tumors, leading to insufficient tumor control [8,9]. To deliver a sufficient dose to such tumors, the tandem and ovoid applicator can be supplemented with a few interstitial needles. In 2011, our group introduced a combination of a few interstitial needles with a Fletcher-Suit Asian Pacific applicator (Elekta, Stockholm, Sweden) under in-room CT guidance [10]. Multiple studies have shown that this combined intracavitary and interstitial (IC/IS) brachytherapy has excellent outcomes for locally advanced disease [7,11]. In addition, brachytherapy applicators equipped with a template for needle insertion have been developed by several groups. In 2006, Dimopoulos et al. introduced the Vienna applicator consisting of a tandem and a ring that enables needle insertion from predefined directions guided through the holes in the ring [12]. In 2009, Jürgenliemk-Schulz et al. introduced the Utrecht applicator consisting of a tandem and two oval ovoids that enable needle insertion from more-varied directions than the Vienna applicator, with the needles guided through the holes in the ovoids [13]. In 2018, Walter et al. introduced the Venezia applicator consisting of a tandem and two lunar-shaped ovoids (that form a ring in combination) functioning as a needle template [14].

The key to the success of IC/IS brachytherapy is to insert interstitial needles in the locations appropriate to achieving a highly conformal dose to the targeted tumor volume. However, the training opportunities for needle application are limited, thereby preventing this technique from becoming widely used by radiation oncologists. To address this issue, this study aimed to develop a training simulator for IC/IS brachytherapy for locally advanced cervical cancer.

## 2. Materials and Methods

### 2.1. Foundation of the Simulator

The foundation of the simulator was developed using transparent acryl to provide visibility. No metal was used to ensure that the simulator was CT and MRI compatible. To enable a physician to approach through the vagina, an acrylic tube mimicking the vagina (50 mm in diameter and 90 mm in length) was installed on an acrylic plate (200 × 100 mm).

### 2.2. Patient-Derived Tumor Phantom

A tumor phantom representing a typical bulky and irregular-shaped tumor was produced. The case on which the tumor phantom was modeled was selected by a review of in-house IGABT records. The inclusion criteria for the review were as follows: (i) patients with newly-diagnosed and pathologically-confirmed cervical cancer treated with definitive radiotherapy using IGABT [7] at Gunma University Hospital (Maebashi, Gunma, Japan) between 2012 and 2020; and (ii) staged as IIIB based on the International Federation of Gynecology and Obstetrics 2009 staging system. For the eligible patients, MRI obtained at the first brachytherapy was inspected by three radiation oncologists (KT, KA, and DI), and ten patients who harbored a bulky tumor with extensive bilateral parametrial involvement were selected. For these ten patients, the high-risk clinical target volume (HR-CTV) was delineated on the T2-weighted MRI according to the recommendations of The Groupe Européen de Curiethérapie and the European Society for Radiotherapy and Oncology [15,16] using MIM Maestro (version 6.8.7, MIM Software Inc., Cleveland, OH, USA). The HR-CTV contour data were then transferred to OsiriX 64-bit (version 12.5.2, Pixmeo, Bernex, Switzerland) and subjected to three-dimensional (3D)-volume rendering. The three radiation oncologists inspected the 3D-reconstructed HR-CTVs from various angles and identified the best case for use as the phantom.

The HR-CTV data of the model patient were then transferred to a computer-aided design software (version 3.0 Creo Parametric, PTC, Boston, MA, USA) and a real-scale 3D volume was exported in acrylonitrile butadiene styrene (ABS) plastic using a 3D printer (Value 3D Magix MF-1100, Mutoh Industries Ltd., Tokyo, Japan). An aluminum mold of the ABS plastic tumor phantom was created using a computerized numerical control milling center (ACCUMILL 4000, DMG Mori Co., Ltd., Nagoya, Aichi, Japan). Finally, a translucent soft silicon tumor phantom was created using the mold.

### 2.3. Trial Use of the IC/IS Training Simulator

Trial use of the developed IC/IS training simulator (consisting of a combination of foundation parts and the tumor phantom) was performed by following the clinical flow of IGABT. An expert or resident radiation oncologist performed the application using either a Fletcher-Suit Asian Pacific applicator or a Venezia applicator, with or without interstitial needles. The expert was a Japanese Radiological Society (JRS) board-certified radiation oncologist with 14 years of clinical experience and more than 800 sessions of IC/IS brachytherapy. The resident was a first-grade JRS resident who had experience in approximately 10 sessions of IC/IS brachytherapy. For the sake of simplicity, the number of needles used was fixed at four. No constraints were set on the insertion locations of the needles. With the Fletcher-Suit Asian Pacific applicator, the needles were inserted in a freehand manner, as described previously [10].

After each application, the expert conducted CT-based treatment planning. CT images were obtained using an in-room scanner and the HR-CTV (i.e., the tumor phantom) was delineated using MIM Maestro. Then, the CT images of the model patient used for the actual first brachytherapy were superimposed on those of each trial application, and the contours of the rectum and bladder in the former were reproduced in each trial application. The contour data were transferred to Oncentra (version 4.5.3, Elekta, Stockholm, Sweden) and treatment plans were created based on a high-dose-rate ^192^Ir remote-after-loading system (microSelectron, Elekta, Stockholm, Sweden) [7]. The highest possible dose was prescribed to the HR-CTV while keeping the dose constraints for the rectum and bladder set at D_2cc_ (i.e., the maximum dose at which any 2 cc of the volume is irradiated) below 6 Gy and 7.6 Gy, respectively; these dose constraints are based on the standard protocol for definitive radiotherapy in Japan that employs a central shielding technique for the later 10–20 Gy of EBRT to the whole pelvis [17,18], where the total equivalent dose in 2 Gy per fraction with an α/β ratio of 3 for D_2cc_ falls broadly within the hard constraints defined in the EMBRACE-II study protocol [19]. D_90_ (i.e., the minimum dose at which any 90% of the volume is irradiated) was utilized as the endpoint of dose coverage to the HR-CTV [18].

## 3. Results

**Figure 1:**
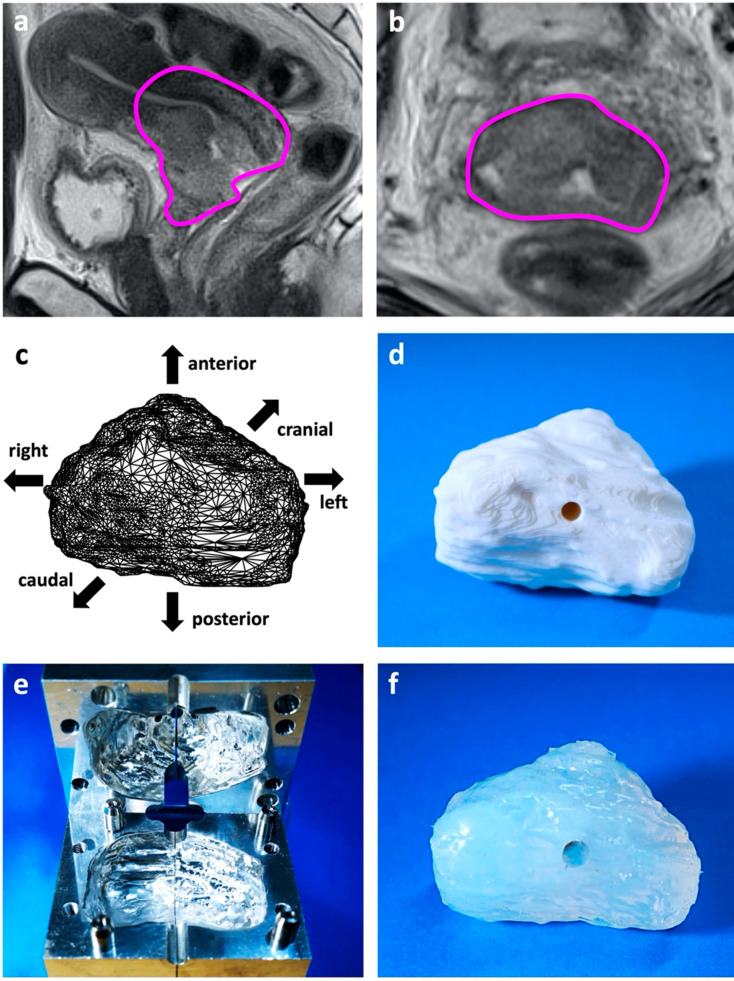
Production of the patient-derived tumor phantom using 3D printing: (**a**) T2-weighted MRI of the model patient obtained at the first brachytherapy session. HR-CTV is delineated in magenta in the sagittal plane and (**b**) axial plane. (**c**) HR-CTV data in computer-aided design software. (**d**) HR-CTV model printed in ABS plastic using a 3D printer. (**e**) Aluminum mold created against the ABS plastic HR-CTV model. (**f**) Translucent soft silicon HR-CTV model created from the aluminum mold.

**Figure 2:**
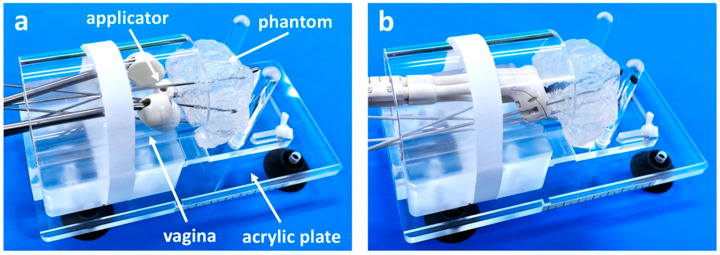
Presentation of the IC/IS training simulator: (**a**) With Fletcher-Suit Asian Pacific applicator and interstitial needles. (**b**) With Venezia applicator and interstitial needles.

In-house IGABT records of 495 eligible patients were reviewed and 70 stage IIIB patients were identified. Detailed inspection of 3D-volume-rendered data led to the identification of the model patient who harbored the HR-CTV at the first brachytherapy of 68 (left–right) × 49 (anterior–posterior) × 45 (cranial–caudal) mm in size (Figure 1a,b, Supplementary Materials Data S1). Using the HR-CTV data, the life-size tumor phantom was created (Figure 1c–f). Finally, the IC/IS training simulator was developed by combining the tumor phantom with the acrylic foundation parts (Figure 2a,b).

**Figure 3:**
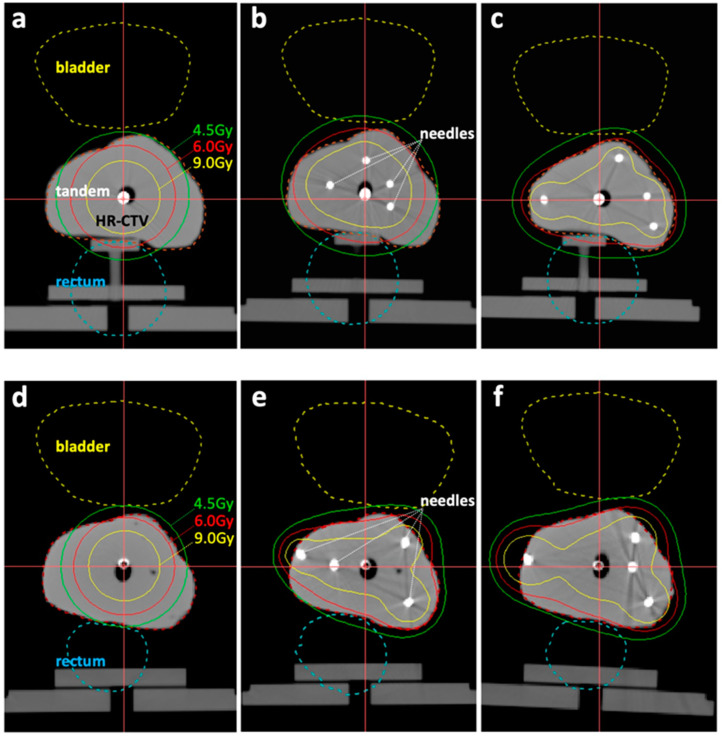
Representative dose-distributions for the treatment plans in the trial use of the IC/IS training simulator with: (**a**) Fletcher-Suit Asian Pacific applicator inserted by an expert; (**b**) Fletcher-Suit Asian Pacific applicator and interstitial needles inserted by the resident; (**c**) Fletcher-Suit Asian Pacific applicator and interstitial needles inserted by an expert; (**d**) Venezia applicator inserted by an expert; (**e**) Venezia applicator and interstitial needles inserted by the resident; or (**f**) Venezia applicator and interstitial needles inserted by expert.

In accord with the clinical flow, the trial use of the developed IC/IS training simulator was performed by the two physicians with different levels of IGABT skills (an expert and a resident). Two applicators (a Fletcher-Suit Asian Pacific applicator and a Venezia applicator) were used with or without interstitial needles. The needles were nicely inserted into the tumor phantom via a vagina-mimicking tube with high visibility (Figure 2a,b), followed by the successful creation of CT-based treatment plans. The Fletcher-Suit Asian Pacific applicator inserted by the expert resulted in an unacceptably low HR-CTV D_90_ of 4.23 Gy (Table 1; Figure 3a). The addition of four needles improved the HR-CTV D_90_ up to 5.69 Gy (by the resident) or 6.70 Gy (by the expert) (Table 1; Figure 3b,c). These trends in dose-volume parameters were broadly consistent when the Venezia applicator was used (Table 1; Figure 3d–f).

## 4. Discussion

Despite its efficacy, multiple reports suggest that the use of brachytherapy in cervical cancer treatment has been diminishing in recent decades. In 2013, Han et al. reported a decrease in the brachytherapy utilization rate from 83% in 1988 to 58% in 2009, using data from the National Cancer Institute’s Surveillance, Epidemiology, and End Results program covering 7359 patients with stage IB2 to IVA cervical cancer who were treated with EBRT [20]. In their study, the use of brachytherapy was independently associated with better cause-specific and overall survival. More recently, Robin et al. reported that less than 50% of cervical cancer patients diagnosed between 2004 and 2012 received a standard of care including EBRT and brachytherapy according to the National Cancer Database [21]. More importantly, the number of brachytherapies performed by residents decreased over the period 2006–2007 to 2010–2011 according to the Accreditation Council of Graduate Medical Education final resident case logs, where the number of interstitial procedures decreased by 25% over the period [22]. These data highlight an increasing need for the education and training of IC/IS brachytherapy, especially for early-career physicians.

Accumulating evidence suggests the efficacy of IC/IS brachytherapy for locally advanced cervical cancers. In a large-scale retrospective study (retroEMBRACE), Fokdal et al. reported that IC/IS brachytherapy resulted in significantly greater HR-CTV D_90_ compared with intracavitary brachytherapy alone, with no difference in doses to organs at risk [23]. In their study, the 3-year local control rate for patients with an HR-CTV > 30 cm^3^ was significantly greater when they were treated with IC/IS brachytherapy. More recently, in a multicenter prospective observational study (EMBRACE-I), IC/IS brachytherapy was used in 43.0% of 1416 cervical cancers, and the overall 5-year local control was 92% [6]. In addition, a sub-group analysis of this cohort reported that IC/IS brachytherapy was used for 69.8% of the cases with bladder wall infiltration [24]. Consistent with these studies, we found in the present study that treatment plans with a tandem and ovoids without interstitial needles resulted in an insufficient dose to a bulky HR-CTV model (approximately 70 cc), whereas the addition of interstitial needles contributed to an HR-CTV D_90_ greater than 6 Gy regardless of applicator type. It is worthy to note that an ongoing multicenter prospective interventional study (EMBRACE-II) employs stricter dose constraints than those for the previous studies [25]. These data suggest high demand for the IC/IS technique in the near future. From this standpoint, the training simulator developed in the present study can contribute to the radiation oncology community by supporting education and dissemination of the IC/IS technique.

Optimization of the intratumoral needle location is important for broadening the therapeutic window of IC/IS brachytherapy [26]. Freehand needle application has the advantage of a high degree of freedom in needle position and direction; however, this, in turn, has the disadvantage of poor reproducibility and difficulty in educating beginners. By contrast, the use of a template for needle application (e.g., the Vienna, Utrecht, and Venezia applicator) provides high reproducibility at the cost of reduced degrees of freedom in needle direction [12,13,14]. To date, no clear indication has been established as to the case types most suited to either the freehand method or templates. In this study, the HR-CTV D_90_ of the plan with freehand needles exceeded that with the Venezia template when the application was performed by the expert. By contrast, the HR-CTV D_90_ of the Venezia-based plan outperformed the freehand-based plan when the application was performed by the resident. These findings may indicate the presence of applicator- or practitioner-specific caveats when educating practitioners in needle insertion.

The training simulator developed in the present study can be easily replicated from the one-off aluminum mold, which will enable inter-practitioner, -modality, and -institute comparisons of IGABT simulation under standardized settings in the future. We can also use this simulator to examine improvements in the performance of the same practitioner or institute. The 3D-tumor printing pipeline reported in this study is applicable to the production of tumor phantoms tailored to individual patients, which could be used in pre-treatment or post-treatment evaluation of IGABT plans in routine clinical practice. Furthermore, 3D-printing technologies can be used in combination with emerging artificial intelligence to improve brachytherapy outcomes [27].

The following limitations to the present study should be noted. First, we developed only one tumor phantom and have reported the results of trial use by only two practitioners, an expert and a resident. The feasibility of the training simulator for educating practitioners in IC/IS brachytherapy should be investigated further by employing multiple phantoms modeled on various irregular-shaped tumors and procedures performed by a greater number of practitioners. Second, we did not assess the dose to the bowel, another organ at risk in IGABT for cervical cancer, and this should be pursued in future studies.

## 5. Conclusions

To support IC/IS brachytherapy training for bulky and irregular-shaped cervical cancers, we developed a training simulator consisting of a patient-derived soft silicon tumor phantom and an acrylic tube mimicking the vagina. In trial use by two physicians with different levels of IGABT skills, a Fletcher-Suit Asian Pacific applicator, and a Venezia applicator with interstitial needles were nicely applied to the simulator, facilitating successful creation of CT-based treatment plans consistent with clinical practice. Further evaluation employing a greater number of phantoms and practitioners is warranted to confirm its educational value.

## Figures and Tables

**Table 1:** Dose-volume parameters for the treatment plans in the trial use of the IC/IS training simulator.

Applicator	Needles	Practitioner	HR-CTV D_90_	Rectum D_2cc_	Bladder D_2cc_
Fletcher-Suit	No	Expert	4.23 Gy	5.99 Gy	3.73 Gy
Fletcher-Suit	Yes	Resident	5.69 Gy	5.99 Gy	4.11 Gy
Fletcher-Suit	Yes	Expert	6.70 Gy	5.98 Gy	4.05 Gy
Venezia	No	Expert	4.16 Gy	5.99 Gy	3.55 Gy
Venezia	Yes	Resident	6.20 Gy	5.99 Gy	3.61 Gy
Venezia	Yes	Expert	6.45 Gy	5.99 Gy	3.83 Gy

The highest possible dose was prescribed to the HR-CTV while keeping the dose constraints for the rectum and bladder set at D_2cc_ below 6 Gy and 7.6 Gy. Fletcher-Suit, Fletcher-Suit Asian Pacific applicator.

## Data Availability

Data is contained within the article.

## Supplementary Materials

The following supporting information can be downloaded at: https://www.mdpi.com/article/10.3390/jcm11113103/s1. STL-format 3D data of the phantom tumor. Drag-and-drop of the tumor allows inspection from any angle.
